# Supplementary material for: Broad protection against clade 1 sarbecoviruses after a single immunization with cocktail spike-protein-nanoparticle vaccine
Source: Nat Commun. 2024 Feb 12;15:1284. doi: 10.1038/s41467-024-45495-6 (PMC10861510; doi:10.1038/s41467-024-45495-6)
Supplement: Supplementary file 1 — Supplementary Information [file 41467_2024_45495_MOESM1_ESM.pdf]

Broad Protection Against Clade 1 Sarbecoviruses After a Single Immunization with Cocktail Spike-Protein-Nanoparticle Vaccine

Peter J. Halfmann[1,†], Kathryn Loeffler[2,†], Augustine Duffy[2,†], Makoto Kuroda[1], Jie E. Yang[3,4,5], Elizabeth R. Wright[3,4,5], Yoshihiro Kawaoka[1,6,7,8]*, Ravi S. Kane[2,9]*

[1]Influenza Research Institute, Department of Pathobiological Sciences, School of Veterinary Medicine, University of Wisconsin, Madison, WI, 53711, USA

[2]School of Chemical & Biomolecular Engineering, Georgia Institute of Technology, Atlanta, Georgia, 30332, USA

[3]Department of Biochemistry, University of Wisconsin, Madison, WI, 53706, USA

[4]Cryo-EM Research Center, Department of Biochemistry, University of Wisconsin, Madison, WI, 53706, USA

[5]Midwest Center for Cryo-Electron Tomography, Department of Biochemistry, University of Wisconsin, Madison, WI, 53706, USA

[6]Division of Virology, Department of Microbiology and Immunology, Institute of Medical Science, University of Tokyo, Tokyo 108-8639, Japan

[7]The Research Center for Global Viral Diseases, National Center for Global Health and Medicine Research Institute, Tokyo 162-8655, Japan.

[8]Pandemic Preparedness, Infection and Advanced Research Center (UTOPIA), University of Tokyo, Tokyo 162-8655, Japan

[9]Wallace H. Coulter Department of Biomedical Engineering, Georgia Institute of Technology, Atlanta, Georgia, 30332, USA

[†] These authors contributed equally

* To whom correspondence should be addressed:

yoshihiro.kawaoka@wisc.edu, ravi.kane@chbe.gatech.edu

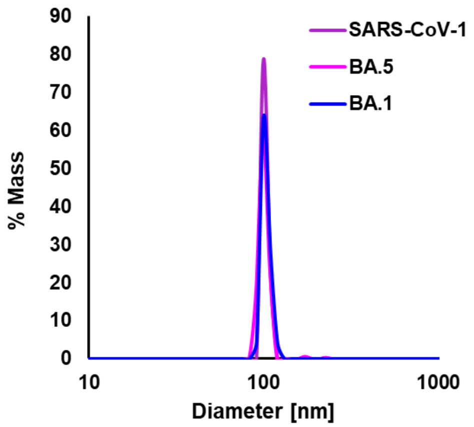

Supplementary Figure 1. Characterization of VLP-SARS-CoV-1-S, VLP-BA.1-S, and VLP-BA.5-S by dynamic light scattering. Colors indicate which VLP-S was measured (SARS-CoV-1: purple; BA.5: magenta; BA.1: dark blue).

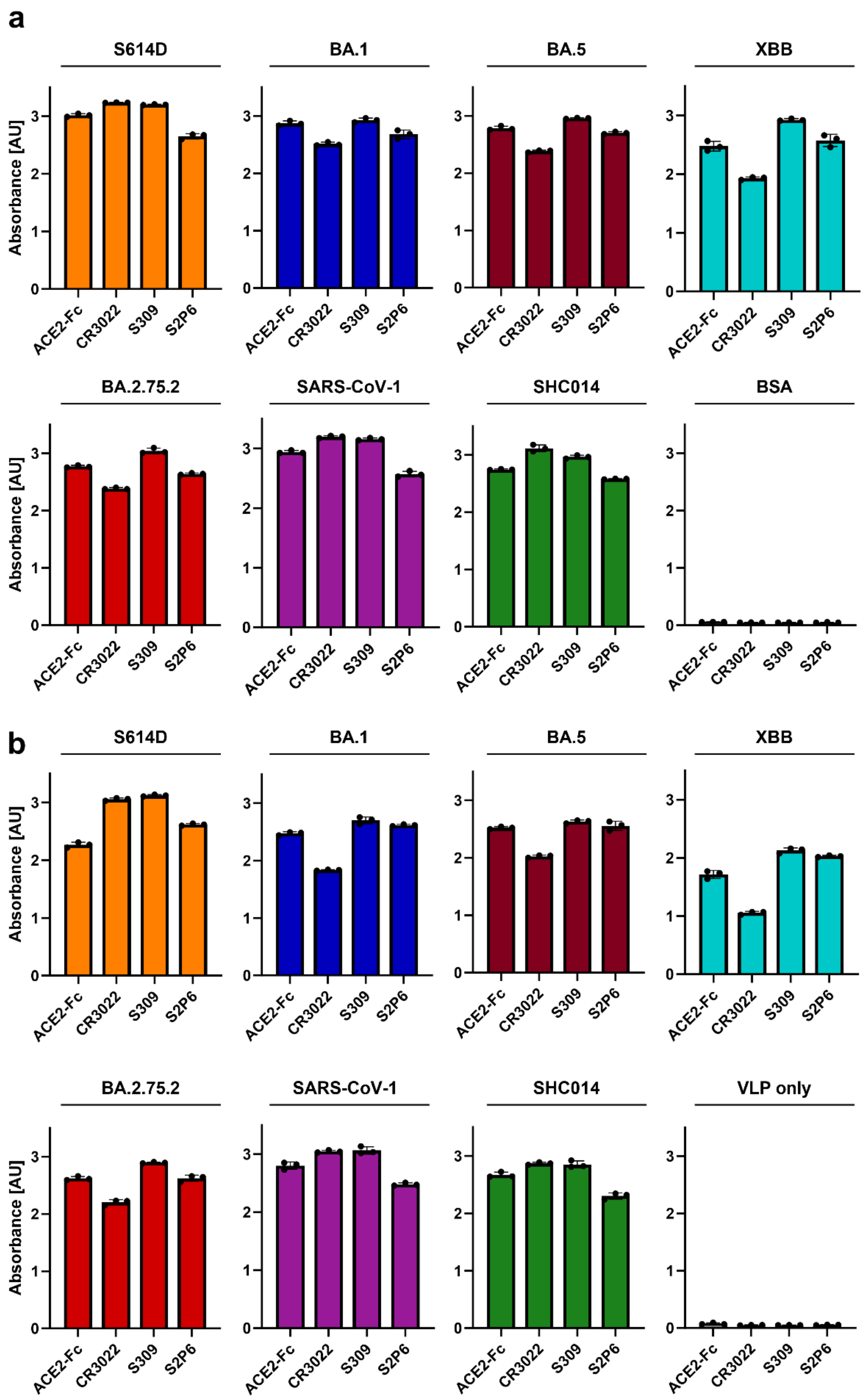

Supplementary Figure 2. Characterization of the binding of ACE2-Fc and S-binding antibodies to (**a**) S proteins and (**b**) VLP-S. (mean ± SD, n = 3: one independent assay with three technical replicates). Colors indicate spike proteins (614D: orange; BA.1: dark blue; BA.5: brown; XBB: cyan; BA.2.75.2: red; SARS-CoV-1: purple; SHC014: green).

a 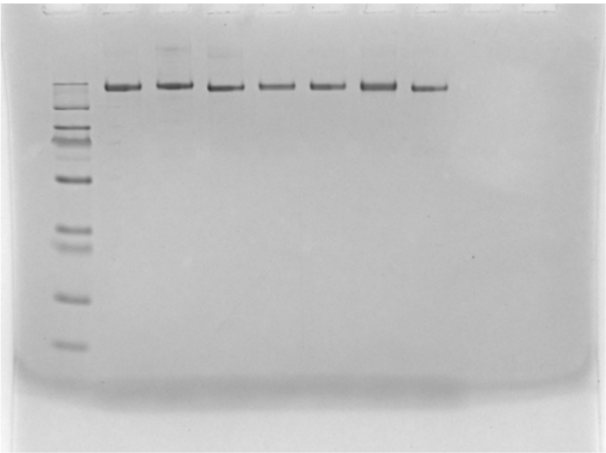 **b** 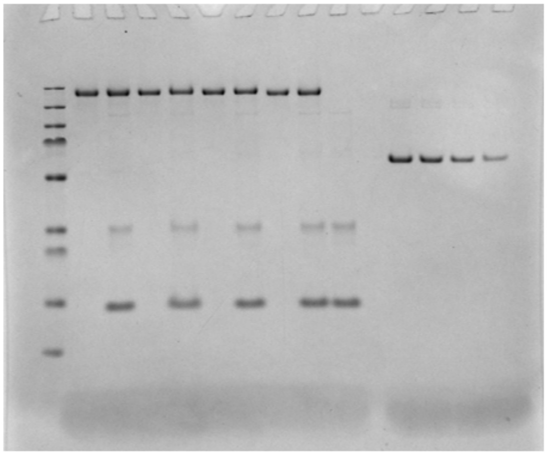

Supplementary Figure 3. Unprocessed SDS-PAGE gel images, cropped versions of which appear in (**a**) Fig. 2a and (**b**) Fig. 2c. These gels were run twice from the same preparation for each sample with similar results.

Supplementary Table 1. Protein sequences

**MS2-AviTag**	MASNFTQFVLVDNGGTGDVTVAPSNFANGVAEWISSNSRSQAYKVTCSVRQSSAQNRK<br>YTIKVEVPKVATQTVGGVELPVAAWRSYLNMELTIPIFATNSDCELIVKAMQGLLKDGN<br>PIPSAIAANSGIYASNFTQFVLVDNGGGLNDIFEAQKIEWHETGDVTVAPSNFANGVAE<br>WISSNSRSQAYKVTCSVRQSSAQNRKYTIKVEVPKVATQTVGGVELPVAAWRSYLNME<br>LTIPIFATNSDCELIVKAMQGLLKDGNPIPSAIAANSGIY
**SARS-CoV-2 HexaPro S**	MFVFLVLLPLVSSQCVNLTTRTQLPPAYTNSFTRGVYYPDKVFRSSVLHSTQDLFLPFFS<br>NVTWFHAIHVSGTNGTKRFDNPVLPFNDGVYFASTEKSNIIRGWIFGTTLDSKTQSLLIV<br>NNATNVVIKVCEFQFCNDPFLGVYYHKNNKSWMESEFRVYSSANNCTFEYVSQPFLMD<br>LEGKQGNFKNLREFVFKNIDGYFKIYSKHTPINLVRDLPQGFSALEPLVDLPIGINITRFQT<br>LLALHRSYLTPGDSSSGWTAGAAAYYVGYLQPRTFLLKYNENGTITDAVDCALDPLSET<br>KCTLKSFTVEKGIYQTSNFRVQPTESIVRFPNITNLCPFGEVFNATRFASVYAWNRKRISN<br>CVADYSVLYNSASFSTFKCYGVSPTKLNDLCFTNVYADSFVIRGDEVRQIAPGQTGKIA<br>DYNYKLPDDFTGCVIAWNSNNLDSKVGGNYNYLYRLFRKSNLKPFERDISTEIYQAGST<br>PCNGVEGFNCYFPLQSYGFQPTNGVGYQPYRVVVLSFELLHAPATVCGPKKSTNLVKN<br>KCVNFNFNGLTGTGVLTESNKKFLPFQQFGRDIADTTDAVRDPQTLEILDITPCSFGGVS<br>VITPGTNTSNQVAVLYQDVNCTEVPVAIHADQLTPTWRVYSTGSNVFQTRAGCLIGAEH<br>VNNSYECDIPIGAGICASYQTQTNSPGSASSVASQSIIAYTMSLGAENSVAYSNNSIAIPTN<br>FTISVTTEILPVSMTKTSVDCTMYICGDSTECSNLLLQYGSFCTQLNRALTGIAVEQDKNT<br>QEVFAQVKQIYKTPPIKDFGGFNFSQILPDPSKPSKRSPIEDLLFNKVTLADAGFIKQYGD<br>CLGDIAARDLICAQKFNGLTVLPPLLTDEMIAQYTSALLAGTITSGWTFGAGPALQIPFP<br>MQMAYRFNGIGVTQNVLYENQKLIANQFNSAIGKIQDSLSSTPSALGKLQDVVNQNAQ<br>ALNTLVKQLSSNFGAISSVLNDILSRLDPPEAEVQIDRLITGRLQSLQTYVTQQLIRAAEIR<br>ASANLAATKMSECVLGQSKRVDFCGKGYHLMSFPQSAPHGVVFLHVTYVPAQEKNFT<br>TAPAICHDGKAHFPREGVFVSNGTHWFVTQRNFYEPQIITTDNTFVSGNCDVVIGIVNNT<br>VYDPLQPELDSFKEELDKYFKNHTSPDVDLGDISGINASVVNIQKEIDRLNEVAKNLNES<br>LIDLQELGKYEQGSGYIPEAPRDGQAYVRKDGEWVLLSTFLGGLNDIFEAQKIEWHEHH<br>HHHH
**BA.1 HexaPro S**	MFVFLVLLPLVSSQCVNLTTRTQLPPAYTNSFTRGVYYPDKVFRSSVLHSTQDLFLPFFS<br>NVTWFHVISGTNGTKRFDNPVLPFNDGVYFASIEKSNIIRGWIFGTTLDSKTQSLLIVNNA<br>TNVVIKVCEFQFCNDPFLDHKNNKSWMESEFRVYSSANNCTFEYVSQPFLMDLEGKQG<br>NFKNLREFVFKNIDGYFKIYSKHTPIIVREPEDLPQGFSALEPLVDLPIGINITRFQTLLALH<br>RSYLTPGDSSSGWTAGAAAYYVGYLQPRTFLLKYNENGTITDAVDCALDPLSETKCTLK<br>SFTVEKGIYQTSNFRVQPTESIVRFPNITNLCPFDEVFNATRFASVYAWNRKRISNCVAD<br>YSVLYNLAPFFTFKCYGVSPTKLNDLCFTNVYADSFVIRGDEVRQIAPGQTGNIADYNY<br>KLPDDFTGCVIAWNSNKLDSKVSGNYNYLYRLFRKSNLKPFERDISTEIYQAGNKPCNG<br>VAGFNCYFPLRSYSFRPTYGVGHQPYRVVVLSFELLHAPATVCGPKKSTNLVKNKCVN<br>FNFNGLKGTGVLTESNKKFLPFQQFGRDIADTTDAVRDPQTLEILDITPCSFGGVSVITPG<br>TNTSNQVAVLYQGVNCTEVPVAIHADQLTPTWRVYSTGSNVFQTRAGCLIGAEYVNNS<br>YECDIPIGAGICASYQTQTKSHGSASSVASQSIIAYTMSLGAENSVAYSNNSIAIPTNFTIS<br>VTTEILPVSMTKTSVDCTMYICGDSTECSNLLLQYGSFCTQLKRALTGIAVEQDKNTQEV<br>FAQVKQIYKTPPIKYFGGFNFSQILPDPSKPSKRSPIEDLLFNKVTLADAGFIKQYGDCLG<br>DIAARDLICAQKFKGLTVLPPLLTDEMIAQYTSALLAGTITSGWTFGAGPALQIPFPMQM<br>AYRFNGIGVTQNVLYENQKLIANQFNSAIGKIQDSLSSTPSALGKLQDVVNHNAQALNT<br>LVKQLSSKFGAISSVLNDIFSRLDPPEAEVQIDRLITGRLQSLQTYVTQQLIRAAEIRASAN<br>LAATKMSECVLGQSKRVDFCGKGYHLMSFPQSAPHGVVFLHVTYVPAQEKNFTTAPAI<br>CHDGKAHFPREGVFVSNGTHWFVTQRNFYEPQIITTDNTFVSGNCDVVIGIVNNTVYDP<br>LQPELDSFKEELDKYFKNHTSPDVDLGDISGINASVVNIQKEIDRLNEVAKNLNESLIDLQ<br>ELGKYEQGSGYIPEAPRDGQAYVRKDGEWVLLSTFLGGLNDIFEAQKIEWHEHHHHHH

**BA.5 HexaPro S**	MFVFLVLLPLVSSQCVNLITRTQSYTNSFTRGVYYPDKVFRSSVLHSTQDLFLPFFSNVT<br>WFHAISGTNGIKRFDNPVLPFNDGVYFASTEKSNIIRGWIFGTTLDSKTQSLLIVNNATNV<br>VIKVCEFQFCNDPFLDVYYHKNNKSWMESEFRVYSSANNCTFEYVSQPFLMDLEGKQG<br>NFKNLREFVFKNIDGYFKIYSKHTPINLGRDLPQGFSALEPLVDLPIGINITRFQTLLALHR<br>SYLTPGDSSSGWTAGAAAYYVGYLQPRTFLLKYNENGTITDAVDCALDPLSETKCTLKS<br>FTVEKGIYQTSNFRVQPTESIVRFPNITNLCPFDEVFNATRFASVYAWNRKRISNCVADY<br>SVLYNFAPFFAFKCYGVSPTKLNDLCFTNVYADSFVIRGNEVSQIAPGQTGNIADYNYK<br>LPDDFTGCVIAWNSNKLDSKVGGNYNYRYRLFRKSNLKPFERDISTEIYQAGNKPCNGV<br>AGVNCYFPLQSYGFRPTYGVGHQPYRVVVLSFELLHAPATVCGPKKSTNLVKNKCVNF<br>NFNGLTGTGVLTESNKKFLPFQQFGRDIADTTDAVRDPQTLEILDITPCSFGGVSVITPGT<br>NTSNQVAVLYQGVNCTEVPVAIHADQLTPTWRVYSTGSNVFQTRAGCLIGAEYVNNSY<br>ECDIPIGAGICASYQTQTKSHGSASSVASQSIIAYTMSLGAENSVAYSNNSIAIPTNFTISV<br>TTEILPVSMTKTSVDCTMYICGDSTECSNLLLQYGSFCTQLKRALTGIAVEQDKNTQEVF<br>AQVKQIYKTPPIKYFGGFNFSQILPDPSKPSKRSPIEDLLFNKVTLADAGFIKQYGDCLGD<br>IAARDLICAQKFNGLTVLPPLLTDEMIAQYTSALLAGTITSGWTFGAGPALQIPFPMQMA<br>YRFNGIGVTQNVLYENQKLIANQFNSAIGKIQDSLSSTPSALGKLQDVVNHNAQALNTL<br>VKQLSSKFGAISSVLNDILSRLDPPEAEVQIDRLITGRLQSLQTYVTQQLIRAAEIRASANL<br>AATKMSECVLGQSKRVDFCGKGYHLMSFPQSAPHGVVFLHVTYVPAQEKNFTTAPAIC<br>HDGKAHFPREGVFVSNGTHWFVTQRNFYEPQIITTDNTFVSGNCDVVIGIVNNTVYDPL<br>QPELDSFKEELDKYFKNHTSPDVDLGDISGINASVVNIQKEIDRLNEVAKNLNESLIDLQE<br>LGKYEQGSGYIPEAPRDGQAYVRKDGEWVLLSTFLGGLNDIFEAQKIEWHEHHHHHH
**XBB HexaPro S**	MFVFLVLLPLVSSQCVNLITRTQSYTNSFTRGVYYPDKVFRSSVLHSTQDLFLPFFSNVT<br>WFHAIHVSGTNGTKRFDNPALPFNDGVYFASTEKSNIIRGWIFGTTLDSKTQSLLIVNNA<br>TNVVIKVCEFQFCNDPFLDVYQKNNKSWMESEFRVYSSANNCTFEYVSQPFLMDLEGK<br>EGNFKNLREFVFKNIDGYFKIYSKHTPINLERDLPQGFSALEPLVDLPIGINITRFQTLLAL<br>HRSYLTPGDSSSGWTAGAAAYYVGYLQPRTFLLKYNENGTITDAVDCALDPLSETKCTL<br>KSFTVEKGIYQTSNFRVQPTESIVRFPNITNLCPFHEVFNATTFASVYAWNRKRISNCVA<br>DYSVIYNFAPFFAFKCYGVSPTKLNDLCFTNVYADSFVIRGNEVSQIAPGQTGNIADYNY<br>KLPDDFTGCVIAWNSNKLDSKPSGNYNYLYRLFRKSKLKPFERDISTEIYQAGNKPCNG<br>VAGSNCYSPLQSYGFRPTYGVGHQPYRVVVLSFELLHAPATVCGPKKSTNLVKNKCVN<br>FNFNGLTGTGVLTESNKKFLPFQQFGRDIADTTDAVRDPQTLEILDITPCSFGGVSVITPG<br>TNTSNQVAVLYQGVNCTEVPVAIHADQLTPTWRVYSTGSNVFQTRAGCLIGAEYVNNS<br>YECDIPIGAGICASYQTQTKSHGSASSVASQSIIAYTMSLGAENSVAYSNNSIAIPTNFTIS<br>VTTEILPVSMTKTSVDCTMYICGDSTECSNLLLQYGSFCTQLKRALTGIAVEQDKNTQEV<br>FAQVKQIYKTPPIKYFGGFNFSQILPDPSKPSKRSPIEDLLFNKVTLADAGFIKQYGDCLG<br>DIAARDLICAQKFNGLTVLPPLLTDEMIAQYTSALLAGTITSGWTFGAGPALQIPFPMQM<br>AYRFNGIGVTQNVLYENQKLIANQFNSAIGKIQDSLSSTPSALGKLQDVVNHNAQALNT<br>LVKQLSSKFGAISSVLNDILSRLDPPEAEVQIDRLITGRLQSLQTYVTQQLIRAAEIRASAN<br>LAATKMSECVLGQSKRVDFCGKGYHLMSFPQSAPHGVVFLHVTYVPAQEKNFTTAPAI<br>CHDGKAHFPREGVFVSNGTHWFVTQRNFYEPQIITTDNTFVSGNCDVVIGIVNNTVYDP<br>LQPELDSFKEELDKYFKNHTSPDVDLGDISGINASVVNIQKEIDRLNEVAKNLNESLIDLQ<br>ELGKYEQGSGYIPEAPRDGQAYVRKDGEWVLLSTFLGGLNDIFEAQKIEWHEHHHHHH

**BA.2.75.2 HexaPro S**	MFVFLVLLPLVSSQCVNLITRTQSYTNSFTRGVYYPDKVFRSSVLHSTQDLFLPFFSNVT WFHAIHVSGTNGTKRFDNPVLPFNDGVYFASTEKSNIIRGWIFGTTLDSKTQSLLIVNNA TNVVIKVCEFQFCNDPFLDVYYHENNKSRMESELRVYSSANNCTFEYVSQPFLMDLEG KQGNFKNLREFVFKNIDGYFKIYSKHTPVNLGRDLPQGFSALEPLVDLPIGINITRFQTLL ALHRSYLTPGDSSSSWTAGAAAYYVGYLQPRTFLLKYNENGTITDAVDCALDPLSETKC TLKSFTVEKGIYQTSNFRVQPTESIVRFPNITNLCPFHEVFNATTFASVYAWNRKRISNCV ADYSVLYNFAPFFAFKCYGVSPTKLNDLCFTNVYADSFVIRGNEVSQIAPGQTGNIADY NYKLPDDFTGCVIAWNSNKLDSKVSGNYNYLYRLFRKSKLKPFERDISTEIYQAGNKPC NGVAGSNCYFPLQSYGFRPTYGVGHQPYRVVVLSFELLHAPATVCGPKKSTNLVKNKC VNFNFNGLTGTGVLTESNKKFLPFQQFGRDIADTTDAVRDPQTLEILDITPCSFGGVSVIT PGTNTSNQVAVLYQGVNCTEVPVAIHADQLTPTWRVYSTGSNVFQTRAGCLIGAEYVN NSYECDIPIGAGICASYQTQTKSHGSASSVASQSIIAYTMSLGAENSVAYSNNSIAIPTNFT ISVTTEILPVSMTKTSVDCTMYICGDSTECSNLLLQYGSFCTQLKRALTGIAVEQDKNTQ EVFAQVKQIYKTPPIKYFGGFNFSQILPDPSKPSKRSPIEDLLFNKVTLADAGFIKQYGDC LGDIAARDLICAQKFNGLTVLPPLLTDEMIAQYTSALLAGTITSGWTFGAGPALQIPFPM QMAYRFNGIGVTQNVLYENQKLIANQFNSAIGKIQDSLSSTPSALGKLQDVVNHNAQAL NTLVKQLSSKFGAISSVLNDILSRLDPPEAEVQIDRLITGRLQSLQTYVTQQLIRAAEIRAS ANLAATKMSECVLGQSKRVDFCGKGYHLMSFPQSAPHGVVFLHVTYVPAQEKNFTTA PAICHDGKAHFPREGVFVSNGTHWFVTQRNFYEPQIITTDNTFVSGNCDVVIGIVNNTVY DPLQPELDSFKEELDKYFKNHTSPDVDLGDISGINASVVNIQKEIDRLNEVAKNLNESLIN LQELGKYEQGSGYIPEAPRDGQAYVRKDGEWVLLSTFLGGLNDIFEAQKIEWHEHHHH HH
**SARS-CoV-1 HexaPro S**	MFIFLLFLTLTSGSDLDRCTTFDDVQAPNYTQHTSSMRGVYYPDEIFRSDTLYLTQDLFL PFYSNVTGFHTINHTFGNPVIPFKDGIYFAATEKSNVVRGWVFGSTMNNKSQSVIIINNST NVVIRACNFELCDNPFFAVSKPMGTQTHTMIFDNAFNCTFEYISDAFSLDVSEKSGNFKH LREFVFKNKDGFLYVYKGYQPIDVVRDLPSGFNTLKPIFKLPLGINITNFRAILTAFSPAQ DIWGTSAAAYFVGYLKPTTFMLKYDENGTITDAVDCSQNPLAELKCSVKSFEIDKGIYQ TSNFRVVPSGDVVRFPNITNLCPFGEVFNATKFPSVYAWERKKISNCVADYSVLYNSTFF STFKCYGVSATKLNDLCFSNVYADSFVVKGDDVRQIAPGQTGVIADYNYKLPDDFMGC VLAWNTRNIDATSTGNYNYKYRYLRHGKLRPFERDISNVPFSPDGKPCTPPALNCYWPL NDYGFYTTTGIGYQPYRVVVLSFELLNAPATVCGPKLSTDLIKNQCVNFNFNGLTGTGV LTPSSKRFQPFQQFGRDVSDFTDSVRDPKTSEILDISPCSFGGVSVITPGTNASSEVAVLY QDVNCTDVSTAIHADQLTPAWRIYSTGNNVFQTQAGCLIGAEHVDTSYECDIPIGAGICA SYHTVGSASSTSQKSIVAYTMSLGADSSIAYSNNTIAIPTNFSISITTEVMPVSMAKTSVD CNMYICGDSTECANLLLQYGSFCTQLNRALSGIAAEQDRNTREVFAQVKQMYKTPTLK YFGGFNFSQILPDPLKPTKRSPIEDLLFNKVTLADAGFMKQYGECLGDINARDLICAQKF NGLTVLPPLLTDDMIAAYTAALVSGTATAGWTFGAGPALQIPFPMQMAYRFNGIGVTQ NVLYENQKQIANQFNKAISQIQESLTTTPTALGKLQDVVNQNAQALNTLVKQLSSNFGA ISSVLNDILSRLDPPEAEVQIDRLITGRLQSLQTYVTQQLIRAAEIRASANLAATKMSECV LGQSKRVDFCGKGYHLMSFPQAAPHGVVFLHVTYVPSQERNFTTAPAICHEGKAYFPR EGVFVFNGTSWFITQRNFFSPQIITTDNTFVSGNCDVVIGIINNTVYDPLQPELDSFKEELD KYFKNHTSPDVDLGDISGINASVVNIQKEIDRLNEVAKNLNESLIDLQELGKYEQGSGYI PEAPRDGQAYVRKDGEWVLLSTFLGGLNDIFEAQKIEWHEHHHHHH

**SHC014 HexaPro S**	MKLLVLVFATLVSSYTIEKCLDFDDRTPPANTQFLSSHRGVYYPDDIFRSNVLHLVQDHF<br>LPFDSNVTRFITFGLNFDNPIIPFRDGIYFAATEKSNVIRGWVFGSTMNNKSQSVIIMNNST<br>NLVIRACNFELCDNPFFVVLKSNNTQIPSYIFNNAFNCTFEYVSKDFNLDLGEKPGNFKD<br>LREFVFRNKDGFLHVYSGYQPISAASGLPTGFNALKPIFKLPLGINITNFRTLLTAFPPRPD<br>YWGTSAAAYFVGYLKPTTFMLKYDENGTITDAVDCSQNPLAELKCSVKSFEIDKGIYQT<br>SNFRVAPSKEVVRFPNITNLCPFGEVFNATTFPSVYAWERKRISNCVADYSVLYNSTSFS<br>TFKCYGVSATKLNDLCFSNVYADSFVVKGDDVRQIAPGQTGVIADYNYKLPDDFLGCV<br>LAWNTNSKDSSTSGNYNYLYRWVRRSKLNPYERDLSNDIYSPGGQSCSAVGPNCYNPL<br>RPYGFFTTAGVGHQPYRVVVLSFELLNAPATVCGPKLSTDLIKNQCVNFNFNGLTGTGV<br>LTPSSKRFQPFQQFGRDVSDFTDSVRDPKTSEILDISPCSFGGVSVITPGTNTSSEVAVLYQ<br>DVNCTDVPVAIHADQLTPSWRVYSTGNNVFQTQAGCLIGAEHVDTSYECDIPIGAGICA<br>SYHTVGSASSTSQKSIVAYTMSLGADSSIAYSNNTIAIPTNFSISITTEVMPVSMAKTSVD<br>CNMYICGDSTECANLLLQYGSFCTQLNRALSGIAVEQDRNTREVFAQVKQMYKTPTLK<br>DFGGFNFSQILPDPLKPTKRSPIEDLLFNKVTLADAGFMKQYGECLGDINARDLICAQKF<br>NGLTVLPPLLTDDMIAAYTAALVSGTATAGWTFGAGPALQIPFPMQMAYRFNGIGVTQ<br>NVLYENQKQIANQFNKAISQIQESLTTTPTALGKLQDVVNQNAQALNTLVKQLSSNFGA<br>ISSVLNDILSRLDPPEAEVQIDRLITGRLQSLQTYVTQQLIRAAEIRASANLAATKMSECV<br>LGQSKRVDFCGKGYHLMSFPQAAPHGVVFLHVTYVPSQERNFTTAPAICHEGKAYFPR<br>EGVFVFNGTSWFITQRNFFSPQIITTDNTFVSGSCDVVIGIINNTVYDPLQPELDSFKEELD<br>KYFKNHTSPDVDLGDISGINASVVNIQKEIDRLNEVAKNLNESLIDLQELGKYEQGSGYI<br>PEAPRDGQAYVRKDGEWVLLSTFLGGLNDIFEAQKIEWHEHHHHHH

Supplementary Table 2: List of coronavirus accession numbers

Clade	Name	Pango Designation	Accession Number	Notes	Reference or Identifying Source
1A	LYRa11	-	AHX37558.1 [https://www.ncbi.nlm.nih.gov/protein/AHX37558.1]		[1]
1A	RsSHC014	-	AGZ48806.1 [https://www.ncbi.nlm.nih.gov/protein/AGZ48806.1]		[2]
1A	WIV1	-	AGZ48828.1 [https://www.ncbi.nlm.nih.gov/protein/AGZ48828.1]		[2]
1A	SARS-CoV-1 Urbani	-	AAP13441.1 [https://www.ncbi.nlm.nih.gov/protein/AAP13441.1]		[3]
1B	Rc-o319	-	BCG66627.1 [https://www.ncbi.nlm.nih.gov/protein/BCG66627.1]		[4]
1B	GD-MP789	-	QIG55945.1 [https://www.ncbi.nlm.nih.gov/protein/QIG55945.1]		[5]
1B	GX-P2V	-	QIQ54048.1 [https://www.ncbi.nlm.nih.gov/protein/QIQ54048.1]		[6]
1B	SARS-CoV-2 Wuhan-Hu-1	B	YP_009724390.1 [https://www.ncbi.nlm.nih.gov/protei	614D	[7]

			n/YP_009724390.1]		
1B	SARS-CoV-2/human/BEL/rega-20174/2021	BA.1	UFO69279.1 [https://www.ncbi.nlm.nih.gov/protein/UFO69279.1]	Omicron	[8]
1B	SARS-CoV-2/human/BHR/22920441474/2022	BA.2.75.2	UVJ48842.1 [https://www.ncbi.nlm.nih.gov/protein/UVJ48842.1]	Omicron	[9]
1B	SARS-CoV-2/human/USA/CA-CDC-STM-MV7TMMQMB/2022	XBB	UZS22117.1 [https://www.ncbi.nlm.nih.gov/protein/UZS22117.1]	Omicron	[10]
1B	SARS-CoV-2/human/USA/JW5734/2022	BA.5.5	UPI46221.1 [https://www.ncbi.nlm.nih.gov/protein/UPI46221.1]	Omicron	[11]
2	HKU3-8	-	ADE34766.1 [https://www.ncbi.nlm.nih.gov/protein/ADE34766.1]		[12]
2	YN2013	-	AIA62330.1 [https://www.ncbi.nlm.nih.gov/protein/AIA62330.1]		[13,14]
2	Cp/Yunnan2011	-	AGC74176.1 [https://www.ncbi.nlm.nih.gov/protein/AGC74176.1]		[13,14,15]
2	ZC45	-	AVP78031.1 [https://www.ncbi.nlm.nih.gov/protein/AVP78031.1]		[16]
3	BM48-31/BGR/2008	-	YP_003858584.1 [https://www.ncbi.nlm.nih.gov/protein/YP_003858584.1]		[17]
3	BtKY72	-	APO40579.1 [https://www.ncbi.nlm.nih.gov/protein/APO40579.1]		[18]
4	RaTG15	-	UFP05053.1 [https://www.ncbi.nlm.nih.gov/protein/UFP05053.1]	Alias of Ra7909	[19]
4	RsYN04	-	QWN56242.1 [https://www.ncbi.nlm.nih.gov/protein/QWN56242.1]		[20]

Supplementary References

1. He, B. et al. Identification of diverse alphacoronaviruses and genomic characterization of a novel severe acute respiratory syndrome-like coronavirus from bats in China. *Journal of Virology* **88,** 7070-7082 (2014).
2. Ge, X.Y. et al. Isolation and characterization of a bat SARS-like coronavirus that uses the ACE2 receptor. *Nature* **503,** 535-538 (2013).
3. Rota, P.A. et al. Characterization of a novel coronavirus associated with severe acute respiratory syndrome. *Science* **300,** 1394-1399 (2003).
4. Murakami, S. et al. Detection and characterization of bat sarbecovirus phylogenetically related to SARS-CoV-2, Japan. *Emerging Infectious Diseases* **26,** 3025-3029 (2020).
5. Liu, P. et al., Are pangolins the intermediate host of the 2019 novel coronavirus (SARS-CoV-2)?. *PLOS Pathogens* **16,** e1008421 (2020).
6. Cao et al., State Key Laboratory of Pathogen and Biosecurity, Beijing Institute of Microbiology and Epidemiology, China. https://www.ncbi.nlm.nih.gov/protein/QIQ54048.1
7. Wu, F. et al. A new coronavirus associated with human respiratory disease in China. *Nature* **579,** 265-269 (2020).
8. Logist, A.-S. et al., Microbiology, Immunology and Transplantation, KU Leuven, Rega Institute, Belgium. https://www.ncbi.nlm.nih.gov/protein/UFO69279.1
9. Alwasti, H., Communicable Disease Laboratory, Public Health Directorate, Bahrain. https://www.ncbi.nlm.nih.gov/protein/UVJ48842.1
10. Howard, D. et al., Respiratory Viruses Branch, Centers for Disease Control and Prevention, USA. https://www.ncbi.nlm.nih.gov/protein/UZS22117.1.
11. Wang, J. et al., Genetics, University of North Carolina at Chapel Hill, USA. https://www.ncbi.nlm.nih.gov/protein/UPI46221.1
12. Lau, S.K. et al. Ecoepidemiology and complete genome comparison of different strains of severe acute respiratory syndrome-related Rhinolophus bat coronavirus in China reveal bats as a reservoir for acute, self-limiting infection that allows recombination events. *Journal of Virology* **84,** 2808-2819 (2010).
13. Wu, Z. et al. ORF8-related genetic evidence for Chinese horseshoe bats as the source of human severe acute respiratory syndrome coronavirus. *The Journal of Infectious Diseases* **213,** 579-583 (2016).
14. Wu, Z. et al. Deciphering the bat virome catalog to better understand the ecological diversity of bat viruses and the bat origin of emerging infectious diseases. *The ISME Journal* **10,** 609-620 (2016).
15. Yang, L. et al. Novel SARS-like betacoronaviruses in bats, China, 2011. *Emerging Infectious Diseases* **19,** 989-991 (2013).
16. Hu, D. et al. Genomic characterization and infectivity of a novel SARS-like coronavirus in Chinese bats. *Emerging Microbes & Infections* **7,** 154 (2018).
17. Drexler, J.F. et al. Genomic characterization of severe acute respiratory syndrome-related coronavirus in European bats and classification of coronaviruses based on partial RNA-dependent RNA polymerase gene sequences. *Journal of Virology* **84,** 11336-11349 (2010).

18. Tao, Y. & Tong, S. Complete genome sequence of a severe acute respiratory syndrome-related coronavirus from Kenyan bats. *Microbiology Resource Announcements* **8,** e00548-19 (2019).
19. Guo, H. et al. Identification of a novel lineage bat SARS-related coronaviruses that use bat ACE2 receptor. *Emerging Microbes & Infections* **10,** 1507-1514 (2021).
20. Zhou, H. et al. Identification of novel bat coronaviruses sheds light on the evolutionary origins of SARS-CoV-2 and related viruses. *Cell* **184,** 4380-4391 (2021).
