# Supplementary material for: Broad protection against clade 1 sarbecoviruses after a single immunization with cocktail spike-protein-nanoparticle vaccine
Source: Nat Commun. 2024 Feb 12;15:1284. doi: 10.1038/s41467-024-45495-6 (PMC10861510; doi:10.1038/s41467-024-45495-6)
Supplement: Supplementary file 3 — Reporting Summary [file 41467_2024_45495_MOESM3_ESM.pdf]

nature portfolio

Corresponding author(s): Yoshihiro Kawaoka, Ravi Kane

Last updated by author(s): 01/19/2024

# Reporting Summary

Nature Portfolio wishes to improve the reproducibility of the work that we publish. This form provides structure for consistency and transparency in reporting. For further information on Nature Portfolio policies, see our Editorial Policies and the Editorial Policy Checklist.

## Statistics

For all statistical analyses, confirm that the following items are present in the figure legend, table legend, main text, or Methods section.

| n/a | Confirmed | |
|---|---|---|
| ☐ | ☒ | The exact sample size (*n*) for each experimental group/condition, given as a discrete number and unit of measurement |
| ☐ | ☒ | A statement on whether measurements were taken from distinct samples or whether the same sample was measured repeatedly |
| ☐ | ☒ | The statistical test(s) used AND whether they are one- or two-sided<br>*Only common tests should be described solely by name; describe more complex techniques in the Methods section.* |
| ☒ | ☐ | A description of all covariates tested |
| ☐ | ☒ | A description of any assumptions or corrections, such as tests of normality and adjustment for multiple comparisons |
| ☐ | ☒ | A full description of the statistical parameters including central tendency (e.g. means) or other basic estimates (e.g. regression coefficient) AND variation (e.g. standard deviation) or associated estimates of uncertainty (e.g. confidence intervals) |
| ☐ | ☒ | For null hypothesis testing, the test statistic (e.g. *F*, *t*, *r*) with confidence intervals, effect sizes, degrees of freedom and *P* value noted<br>*Give P values as exact values whenever suitable.* |
| ☒ | ☐ | For Bayesian analysis, information on the choice of priors and Markov chain Monte Carlo settings |
| ☒ | ☐ | For hierarchical and complex designs, identification of the appropriate level for tests and full reporting of outcomes |
| ☒ | ☐ | Estimates of effect sizes (e.g. Cohen's *d*, Pearson's *r*), indicating how they were calculated |

*Our web collection on statistics for biologists contains articles on many of the points above.*

## Software and code

Policy information about availability of computer code

| | |
|---|---|
| Data collection | Gen5 2.07 (BioTek), Image Lab 5.2.1 (BioRad), Unicorn 7 (Cytiva), SerialEM 3.84 (University of Colorado Boulder), ImmunoSpot S6 Analyzer (Cellular Technology) |
| Data analysis | Clustal Omega 1.2.4 (UCD Dublin), PhyML 3.3.20220408 (University of Montpellier), Prism 9 (Graphpad), Racmacs 1.1.35 (University of Cambridge), R 4.2.1, RStudio 2022.07.1 (Posit), ImmunoCapture software (Cellular Technology), BioSpot (Cellular Technology) |

For manuscripts utilizing custom algorithms or software that are central to the research but not yet described in published literature, software must be made available to editors and reviewers. We strongly encourage code deposition in a community repository (e.g. GitHub). See the Nature Portfolio guidelines for submitting code & software for further information.

## Data

Policy information about availability of data

All manuscripts must include a data availability statement. This statement should provide the following information, where applicable:

- Accession codes, unique identifiers, or web links for publicly available datasets
- A description of any restrictions on data availability
- For clinical datasets or third party data, please ensure that the statement adheres to our policy

We have added a data availability statement to the manuscript: All data supporting the conclusions of this paper can be found within the paper, Supplementary Information, and Source Data file. Protein sequences for MS2-AviTag and the HexaPro S proteins are available in Supplementary Table 1. GenBank and RefSeq accession numbers for Fig. 1 are available in Supplementary Table 2. Structures used to generate Fig. 2b are available from the PDB using accession codes 2MS2

[https://doi.org/10.2210/pdb2MS2/pdb], 3RY2 [https://doi.org/10.2210/pdb3RY2/pdb], and 6VSB [https://doi.org/10.2210/pdb6VSB/pdb]. Unprocessed SDS-PAGE gel images for Fig. 2 are available in Supplementary Figure 3. Source data for Fig. 2e; Supplementary Figure 2; Fig. 3a, 3b, and 3c; Fig. 4; and Fig. 5 are available in the Source Data file.

## Research involving human participants, their data, or biological material

Policy information about studies with human participants or human data. See also policy information about sex, gender (identity/presentation), and sexual orientation and race, ethnicity and racism.

| | |
|---|---|
| Reporting on sex and gender | No human research participants were used in this study. |
| Reporting on race, ethnicity, or other socially relevant groupings | No human research participants were used in this study. |
| Population characteristics | No human research participants were used in this study. |
| Recruitment | No human research participants were used in this study. |
| Ethics oversight | No human research participants were used in this study. |

Note that full information on the approval of the study protocol must also be provided in the manuscript.

# Field-specific reporting

Please select the one below that is the best fit for your research. If you are not sure, read the appropriate sections before making your selection.

[x] Life sciences  [ ] Behavioural & social sciences  [ ] Ecological, evolutionary & environmental sciences

For a reference copy of the document with all sections, see nature.com/documents/nr-reporting-summary-flat.pdf

# Life sciences study design

All studies must disclose on these points even when the disclosure is negative.

| | |
|---|---|
| Sample size | The number of animals used in each experiment was chosen based on our previous studies with SARS-CoV-2 infection of hamsters, in which the sample size was sufficient to evaluate a statistically significant difference between groups. Group sizes were based on our previous vaccine manuscripts with hamsters (Chiba et al., Communications Biology, 2021; Halfmann et al., eBioMedicine, 2022) |
| Data exclusions | No data was excluded. |
| Replication | Virus titer are given for each individual animal as the individual dots of the graph for each vaccine group. Each animal study was performed only once with 3-4 animals in each group given the use of USDA covered species (hamsters). Though each study was not done in duplicate, the same challenge viruses were used in different studies, and the titers of the challenge virus in the tissues of the control animals between different studies were similar attesting to reproducibility. |
| Randomization | All animals were randomly assigned to each experimental group for all the experiments. |
| Blinding | Not relevant for this study; different researchers took part in the different parts of the study (vaccination, challenge, tissue collection, titrations, and analysis). |

# Reporting for specific materials, systems and methods

We require information from authors about some types of materials, experimental systems and methods used in many studies. Here, indicate whether each material, system or method listed is relevant to your study. If you are not sure if a list item applies to your research, read the appropriate section before selecting a response.

## Materials & experimental systems

| n/a | Involved in the study | |
|---|---|---|
| [ ] | [x] | Antibodies |
| [ ] | [x] | Eukaryotic cell lines |
| [x] | [ ] | Palaeontology and archaeology |
| [ ] | [x] | Animals and other organisms |
| [x] | [ ] | Clinical data |
| [x] | [ ] | Dual use research of concern |
| [x] | [ ] | Plants |

## Methods

| n/a | Involved in the study | |
|---|---|---|
| [x] | [ ] | ChIP-seq |
| [x] | [ ] | Flow cytometry |
| [x] | [ ] | MRI-based neuroimaging |

# Antibodies

| | |
|---|---|
| Antibodies used | horseradish peroxidase-conjugated anti-human IgG Fc goat antibody (MP Biomedical, catalog # 674171); 1C7C7 (Sigma-Aldrich, catalog # MA5-29982), HRP-conjugated goat anti-mouse (ThermoFisher, catalog # 31430); ACE2-Fc, CR3022, S309, S2P6 (recombinant, produced in-house) |
| Validation | The anti-human IgG Fc goat antibody is commercially available from MP Biomedical. 1C7C7 is commercially available from Sigma-Aldrich and is validated against SARS-CoV-1 and SARS-CoV-2 nucleoprotein. HRP-conjugated goat anti-mouse is commercially available from ThermoFisher. ACE2-Fc was previously used to characterize the SARS-CoV-2 spike protein in Chiba et al. (2021). CR3022, S209, and S2P6 were validated as active against the SARS-CoV-2 spike protein in references cited in the manuscript -- ter Meulen et al. (2006), Pinto et al. (2020), and Pinto et al. (2021) -- respectively. |

# Eukaryotic cell lines

Policy information about cell lines and Sex and Gender in Research

| | |
|---|---|
| Cell line source(s) | Expi293F cells were supplied by Thermo Fisher Scientific and first described by Jones et al. (DOI: 10.1089/gen.32.17.21). Vero Vero E6 TMPRSS2 cells were obtained from the National Institute of Infectious Diseases in Japan and have been published on. Vero E6 TMPRSS2-T2A-ACE2 cells were obtained from Dr. Barney Graham, NIAID Vaccine Research Center. |
| Authentication | Expi293F, Vero E6-TMPRSS2 and Vero E6 TMPRSS2-T2A-ACE2 cells were not authenticated, but cell morphology, growth, viability, virus plaque morphology, and/or protein expression were consistent with expectations. |
| Mycoplasma contamination | Vero E6 TMPRSS2 and Vero E6 TMPRSS2-T2A-ACE2 cells were tested monthly in our laboratory and were negative each time for mycoplasma contamination. Expi293F cells were not tested for mycoplasma contamination. |
| Commonly misidentified lines (See ICLAC register) | No commonly misidentified lines were used |

# Animals and other research organisms

Policy information about studies involving animals; ARRIVE guidelines recommended for reporting animal research, and Sex and Gender in Research

| | |
|---|---|
| Laboratory animals | 4–5-week-old female Syrian golden hamsters from Envigo and K18-human ACE2 homozygous transgenic hamsters (Golden et al. 2022) from an established colony at UW-Madison (females, 5-6 weeks old) were used in this study. |
| Wild animals | This study did not involve wild animals. |
| Reporting on sex | Hamsters are all females due availability |
| Field-collected samples | This study did not involve samples collected from the field. |
| Ethics oversight | All studies were conducted under an approved protocol reviewed by the Institutional Animal Care and Use Committee at the University of Wisconsin. University of Wisconsin-Madison IACUC protocol # V006426 |

Note that full information on the approval of the study protocol must also be provided in the manuscript.

# Plants

| | |
|---|---|
| Seed stocks | *Report on the source of all seed stocks or other plant material used. If applicable, state the seed stock centre and catalogue number. If plant specimens were collected from the field, describe the collection location, date and sampling procedures.* |
| Novel plant genotypes | *Describe the methods by which all novel plant genotypes were produced. This includes those generated by transgenic approaches, gene editing, chemical/radiation-based mutagenesis and hybridization. For transgenic lines, describe the transformation method, the number of independent lines analyzed and the generation upon which experiments were performed. For gene-edited lines, describe the editor used, the endogenous sequence targeted for editing, the targeting guide RNA sequence (if applicable) and how the editor was applied.* |
| Authentication | *Describe any authentication procedures for each seed stock used or novel genotype generated. Describe any experiments used to assess the effect of a mutation and, where applicable, how potential secondary effects (e.g. second site T-DNA insertions, mosiacism, off-target gene editing) were examined.* |
